# Cultural Adaptation of the Home-Based Tailored Activity Program in Chile

**DOI:** 10.1093/geroni/igaa057.2161

**Published:** 2020-12-16

**Authors:** Jean Gajardo, Jose Aravena, Ignacia Navarrete, Andrea Slachevsky, Laura Gitlin

**Affiliations:** 1 Universidad San Sebastián, Santiago, Chile; 2 School of Public Health, Yale University, New Haven, Connecticut, United States; 3 Universidad de Chile, Santiago, Region Metropolitana, Chile; 4 Universidad de chile, Santiago, Region Metropolitana, Chile; 5 Drexel University, Philadelphia, Pennsylvania, United States

## Abstract

Chile is currently implementing policies addressing dementia care with efforts to translate evidence-based programs towards culturally sensitive models of care. This study describes the cultural adaptation of the Tailored Activity Program (TAP). A complementary mixed-method design was performed following the 4-phase Dynamic Adaptation Process (DAP) model by Aarons et al, 2012. Ten dyads (family caregivers and people with dementia) completed a regular 8-session home-based TAP intervention during 2017-2018. Qualitative data was collected through interviews and observation with caregivers, and weekly follow-up and a focus group with provider occupational therapists. Quantitative data in pilot testing was obtained through assessments at baseline and after intervention. The TAP was well accepted by family caregivers, and sociocultural adaptations on content, context, target level, and training were identified. Significant reduction of frequency and severity of neuropsychiatric symptoms in individuals with dementia was found, and caregivers reported reduction of depressive symptoms, improved perceived well-being & self-confidence. Part of a symposium sponsored by the Behavioral Interventions for Older Adults Interest Group.

